# Genomic and functional dissection of natural transformation-related genes in *Piscirickettsia salmonis*

**DOI:** 10.1128/spectrum.03173-25

**Published:** 2026-02-04

**Authors:** Sebastián Higuera-Llantén, Nicolas Ojeda, Javiera Protz, Sergio H. Marshall

**Affiliations:** 1 Laboratorio de Genética e Inmunología Molecular, Facultad de Ciencias, Instituto de Biología, Pontificia Universidad Católica de Valparaíso, Campus Curauma, Valparaíso, Chile; University of Manitoba, Winnipeg, Manitoba, Canada

**Keywords:** *Piscirickettsia salmonis*, ComEC, competence, natural transformation, functional characterization

## Abstract

*Piscirickettsia salmonis,* the etiological agent of piscirickettsiosis, represents the main health challenge for Chilean salmon farming and an emerging threat to global salmonid production. Inspection of all *P. salmonis* genomes available shows that the *comEC* gene*,* encoding the DNA uptake channel required for natural transformation (NT) in competent bacteria, is interrupted by various transposable elements, whereas other NT determinants (*comEA*, *comFB*, *comM*, *comL/bamD*, and *dprA*) remain intact and highly conserved. Using *P. salmonis* Psal-103 (EM-genogroup) and Psal-104b (LF-genogroup) as representatives of the two most prevalent genogroups in Chile, we combined comparative genomics, gene expression analysis, heterologous expression of *comEC* using homologs from naturally competent bacteria, and CRISPRi-mediated knockdown of the remaining NT-related genes to examine their functionality. According to our results, expression of *comEC* with homologs from *Legionella pneumophila* and *Vibrio cholerae* impaired *P. salmonis* viability in axenic culture, indicating a fitness burden. CRISPRi-mediated repression of *comL/bamD* revealed its essentiality in Psal-104b and a critical role during infection of the SHK-1 cell line in Psal-103. Repression of *comM* tended to reduce cytopathogenicity in both strains, while repression of *comFB* was associated with a modest delay in cytopathic damage in Psal-104b. Repression of *recA* and *comEA* caused moderate reductions in cytopathic activity in Psal-103. By contrast, *dprA* showed no detectable phenotypes. Together, our results indicate that despite the irreversible interruption of *comEC*, *P. salmonis* has retained NT-related genes that contribute to fitness in a gene- and genogroup-specific manner, providing a framework to investigate how determinants of horizontal gene transfer function beyond DNA uptake.

**IMPORTANCE:**

Despite its major impact on salmon aquaculture, *Piscirickettsia salmonis* remains poorly characterized at the functional level, largely due to long-standing limitations in genetic tractability. Here, we implement and combine multiple genetic approaches, including CRISPR interference, site-specific chromosomal integration, and heterologous gene expression, to functionally interrogate natural transformation (NT)-related genes in this pathogen. Using representative strains from the two most prevalent Chilean genogroups, we show that conserved NT-associated genes contribute to bacterial physiology and infection in a genogroup-dependent manner. Moreover, and beyond the specific biological findings, this work establishes a versatile genetic platform for functional studies in *P. salmonis*, expanding the experimental toolbox available to study this pathogen and supporting future efforts aimed at understanding its biology and the development of novel biotechnological approaches.

## INTRODUCTION

*Piscirickettsia salmonis* is a facultative intracellular Gammaproteobacterium and the causative agent of piscirickettsiosis (or SRS, by salmonid rickettsial septicemia), a multisystemic disease that has affected net pen-reared salmonids since the last two decades of the past century (1–6). The bacterium is primarily transmitted through water, where it enters fish through the gills or other mucosal surfaces and causes widespread infections with high mortality if left uncontrolled (6, 7). Chile, which produces around 30% of the world’s farmed salmon (8), has the most serious cases, with estimated annual losses between US$400 and US$700 million and a regime of intensive use of antibiotics as part of sanitary measures (9–11). In addition to its sustained health and economic impact in Chile, the occurrence of sporadic outbreaks in other salmon-producing countries in the Northern Hemisphere (12, 13) underscores its relevance as a pathogen of concern for global salmon aquaculture.

Despite representing a major health and economic challenge in salmon aquaculture, *P. salmonis* remains poorly uncharacterized at the functional level. Indeed, its early study was complicated by a long-standing taxonomic misclassification that placed it as a strictly intracellular *Rickettsia* belonging to the Alphaproteobacteria class (1). This misconception persisted for nearly two decades until phylogenetic analyses reclassified *P. salmonis* as a Gammaproteobacterium. Although still formally assigned to the order Thiotrichales (14), recent phylogenomic studies place Piscirickettsiaceae as a distinct lineage within the Gammaproteobacteria class, indicating a sister relationship to the Legionellales (15, 16), thus phylogenetically distant from regular rickettsias.

This erroneous taxonomic classification hindered early research and delayed the development of appropriate axenic culture methods until the late 2000s (17, 18). Although this bacterium is now recognized for its ability to replicate under diverse conditions, including planktonic growth, biofilm formation, solid media, and the intracellular milieu in host cells (13, 17–19), little is known about how *P. salmonis* orchestrates gene regulation, stress tolerance, virulence traits, or which genes are essential for its survival across these contexts (20). More broadly, while omics analyses have led to the identification of several candidate virulence factors, only a small subset has been functionally validated to date (21, 22). Additionally, over 40% of the annotated coding sequences (CDS) in *P. salmonis* correspond to hypothetical proteins, highlighting the prevalence of genes with no known function and no close homologs in model organisms (13, 16, 23). Notably, recent comparative genomic analyses have also revealed that *P. salmonis* has an unusually high abundance of transposable elements (TEs) (16). These mobile genetic elements (MGEs) may disrupt regulatory networks, obscure ancestral functions, or contribute to gene pseudogenization, further complicating functional inference (24, 25).

A notable example of a potentially lost function is hinted by the perturbation of *comEC*, an essential component of the competence machinery for natural transformation (NT) in bacteria, which is truncated by TEs in all available *P. salmonis* genomes (16). NT is a genetically encoded, tightly regulated mechanism of horizontal gene transfer (HGT) that enables bacteria to acquire extracellular DNA and incorporate it into their genome, either as autonomously replicating elements (e.g., plasmids) or through chromosomal integration by homologous recombination (26–28). This process, therefore, relies on a conserved set of genetic determinants involved in DNA binding, translocation, and recombination (27). In gram-negative bacteria, the initial uptake of double-stranded DNA typically involves surface-associated structures such as type IV pili (T4P) or type II/IV secretion systems (T2SS/T4SS), which facilitate DNA entry into the periplasm—or functional homologs of these proteins in naturally competent gram-positive bacteria (29, 30). Once inside, one strand is degraded and the complementary strand is transported into the cytosol through the ComEC channel, where it is bound by DprA and ultimately recombined via RecA (26, 31–33).

In addition to T4P and T4SS loci (21, 34, 35), multiple other NT-related genes, such as *comEA, comFB, comM, comL*, and *dprA*, remain intact and highly conserved across *P. salmonis* genogroups. Motivated by the conserved interruption of *comEC*, we introduced *comEC* homologs from naturally competent bacteria to evaluate the physiological consequences of their heterologous expression in *P. salmonis*. On the other hand, the preservation of numerous competence-related genes remains intriguing; while their original role in NT may no longer be operative, these genes may still contribute to bacterial physiology through functions independent of HGT. To explore this possibility, we conducted an integrative analysis combining comparative genomics, transcriptional profiling, heterologous expression of *comEC* homologs from naturally competent bacteria, and CRISPRi-mediated repression of conserved NT-related genes to assess their essentiality and contribution to bacterial fitness.

## MATERIAL AND METHODS

### Bacterial strains and culture conditions

*P. salmonis* strains Psal-103 (from the EM genogroup) and Psal-104b (from the LF genogroup) were selected as representatives of the two most prevalent genogroups circulating in Chile. Both strains have been minimally passaged, and their closed high-quality genomes were obtained using a combination of PacBio and Illumina platforms. Routine culture of all *P. salmonis* wild-type and mutant strains was performed on IFOP-agar plates (36), consisting of Trypticase Soy Agar (BD BBL, 40 g/L) and NaCl (15 g/L), supplemented with defibrinated sheep blood (50 mL/L), fetal bovine serum (FBS, 50 mL/L), D-glucose (10 g/L), and L-cysteine (1 g/L). For liquid experiments, bacterial suspensions and washes were carried out using IFOP broth (36), composed of CASO broth (Merck, 25 g/L) and NaCl (15 g/L), supplemented with D-glucose (10 g/L), L-cysteine (1 g/L), and FBS (50 mL/L). All *P. salmonis* strains were stored at −80°C in L-15 medium supplemented with 20% FBS and 10% DMSO. *Escherichia coli* strains were routinely grown in LB medium (solid or liquid) at 37°C and stored at −80°C in liquid LB supplemented with 12% (vol/vol) glycerol. A complete list of the *P. salmonis* strains and mutants used in this study is provided in Table S1.

### Comparative analysis of the *comEC* locus

We analyzed the publicly available *P. salmonis* genomes and extracted the *comEC* loci, including the *comEC*-annotated CDS and intermediate sequences. Manual analysis identified CDS coding for an N-terminal ComEC fragment in the Canadian and EM strains, not recognized as ComEC by the annotation algorithms; these CDS were included on the final *comEC* loci. Nucleotide sequences were also aligned using CAGECAT Clinker to assess synteny (37). To analyze the structure and conservation of the ComEC sequence from *P. salmonis*, we concatenated the ComEC CDS from representative genomes and aligned them using the Alignment Tool from CLC Genomics Workbench 12.0 (QIAGEN). The alignment was further adjusted manually and a Pfam domain search using the Pfam-A v.37.4 database (38) revealed the presence of DUF4131, Competence, and Lactamase_B domains (Fig. S2).

### Comparative analysis of NT-related genes

To assess the conservation of NT-related genes between *P. salmonis* strains from the two most representative genogroups in Chile, nucleotide and amino acid sequences from Psal-103 and Psal-104b were compared using BLAST+ v.2.13.0 (NCBI). For broader taxonomic context, nucleotide and amino acid sequences of these genes from Psal-104b (used as a proxy) were subsequently queried against the NCBI ClusteredNR protein database to identify representative matches across taxa.

### Plasmid construction

Plasmid pJMP1159 (Addgene #19250), containing a mini-Tn7 transposon, was used as the scaffold for both *comEC* heterologous expression and CRISPRi-mediated repression constructs in *P. salmonis*. The mini-Tn7 cassette carries a constitutively expressed *rfp*, a gentamicin resistance marker, and a dCas9-based CRISPRi module regulated by the Plac/LacI system.

For *comEC* heterologous expression, the dCas9 CDS in pJMP1159 was removed by inverse PCR using outward-facing primers and Q5 High-Fidelity DNA Polymerase (NEB). In parallel, *comEC* homologs from *Legionella pneumophila* (GenBank: AOU51326.1), *Vibrio cholerae* (GenBank: QKV04914.1), and *Piscirickettsia litoralis* Y2 (GenBank: ODN41896.1) were PCR-amplified with Q5 High-Fidelity DNA Polymerase using primers with overlapping ends complementary to the linearized vector. Gibson Assembly Master Mix (New England Biolabs) was used to insert the *comEC* homologs into the pJMP1159 backbone. Homolog CDS were purchased by *de novo* synthesis from GenScript (USA), each provided as a pUC18-cloned insert. All primers used for Gibson Assembly and their PCR conditions are detailed in Table S2.

For CRISPRi-mediated repression, the original *rfp*-targeting sgRNA in pJMP1159 was replaced with guides specific to *comEA*, *comFB*, *comM*, *comL* (*bamD*), *dprA*, or *recA* CDS. Guide sequences were introduced by inverse PCR with Q5 High-Fidelity DNA Polymerase using primers encoding the new guide sequences at their 3′ ends and 5′ extensions overlapping the vector backbone. These primers were used in combination with PriCas1 and PriCas2 primers to amplify the plasmid in two overlapping fragments, further assembled through Gibson cloning. The sgRNAs were designed in CRISPy-web using the closed genomes of Psal-103 (RefSeq: GCF_009709455.1) and Psal-104b (RefSeq: GCF_023008185.1), prioritizing the following selection criteria, in order: binding to the non-coding strand, conservation across both strains, proximity to predicted transcription start sites, and minimal predicted off-targets (39). Correct assembly of sgRNA in CRISPRi plasmids and proper *comEC* CDS insertion was confirmed by Sanger sequencing (Macrogen, South Korea). The complete list of sgRNAs used in this study is provided in Table 1. Primers used for Gibson Assembly of CRISPRi constructs and their PCR conditions are also detailed in Table S2.

**TABLE 1:** sgRNAs used for CRISPRi targeting of NT-related genes in *P. salmonis^a^*

Gene product	RefSeq accession		sgRNA
	Psal-103	Psal-104b	
*comFB*	QGP42219.1	UOX25998.1	TAGATTCTCGAGGTCTTCAT
*comL/BamD*	QGP43286.1	UOX24917.1	AGTATCTAGGTCACCTTGTT
*dprA*	QGP44521.1	UOX23705.1	GCTTCTTGACCGGAGGGTGT
*recA*	QGP43128.1	UOX25422.1	ATTGCAGAGTGCCAGAAAAA
*comEA*	QGP43645.1	UOX24559.1	ACCACAGCCATTTAGAACCG
*comM*	QGP44472.1	UOX23752.1	GGCGCATGCTAAGCGCGCTT

^*a*^ Single-guide RNAs (sgRNAs) were designed using the CRISPy-web tool based on the complete genomes of *P. salmonis* strains Psal-103 and Psal-104b. Guides were selected to target conserved regions near the transcription start site on the non-coding strand, ensuring compatibility across both strains, prioritized by minimal off-target potential. All sgRNAs were cloned into the pJMP1159 vector by replacing the original *rfp*-targeting guide using plasmid-reverse PCR with universal primers modified at their 5′ ends to include each sgRNA sequence. Final constructs were transferred to *P. salmonis* via triparental conjugation.

### Construction of *P. salmonis* mutants

Final pJMP1159 derivatives were introduced into *E. coli* S17-1 λ pir via chemical transformation of Mix & Go!-prepared competent cells (Zymo Research). A second *E. coli* S17-1 λ pir strain carrying the helper plasmid pJMP1039 (Addgene #119239), encoding the Tn7 transposase, was included to mediate chromosomal integration of mini-Tn7. Conjugation with *P. salmonis* recipient strains (Psal-103 or Psal-104b) was performed as described in reference 21 through triparental mating. Transconjugants were selected on IFOP-agar plates containing gentamicin (5 μg/mL) to select for mini-Tn7 integration and polymyxin B (1 μg/mL) to counter-select donor strains. Integration at the *attTn7* site was confirmed by colony PCR under standard conditions using GoTaq Green Master Mix (Promega) and strain-specific primers for Psal-103 and Psal-104b (Table S2).

### Serial dilution spot assays on solid medium

Fresh colonies of *P. salmonis* were resuspended in IFOP broth and adjusted to an optical density at 600 nm (OD_600_) of 1.0. *E. coli* colonies were resuspended in 0.85% saline and adjusted to the same OD_600_. Ten-fold serial dilutions (up to 10^−5^) were prepared, and 3 µL of each dilution were spotted onto IFOP agar (*P. salmonis*) or LB agar (*E. coli*) supplemented or not with 0.1 mM IPTG. Plates were incubated at 18°C for 8 days (*P. salmonis*) or at 37°C for 24 h (*E. coli*). All strains, including wild-type and default-vector controls, were tested under induced (0.1 mM IPTG) and non-induced conditions. After incubation, the bacterial growth at each dilution was documented photographically to assess changes in viability. As IPTG induction controls in *E. coli*, we included pJMP1159 and pJMP1159::ilux-carrying strains. Luminescence from ilux constructs was measured using a ChemiDoc system (Bio-Rad).

### Growth curve analysis

*P. salmonis* colonies were resuspended in IFOP broth and adjusted to an initial OD_600_ of 0.02 in a transparent 96-well plate (Nunc, Thermo Fisher Scientific). Cultures were incubated at 18°C for 96 h under static conditions, and OD_600_ was recorded at regular intervals using a SPECTROstar Nano Absorbance Plate Reader (BMG Labtech), with shaking for 30 s at 400 rpm prior to each measurement. *E. coli* cultures were prepared similarly and incubated at 37°C for 24 h. All strains, including wild-type and default-vector controls, were tested under induced (0.1 mM IPTG) and non-induced conditions.

### SHK-1 cell infection

To assess the expression levels of NT-related genes during host interaction, SHK-1 cell infection assays were conducted using wild-type *P. salmonis* strains Psal-103 and Psal-104b. Infection assays were performed using the SHK-1 cell line (ECACC 97111106), derived from *Salmo salar* head kidney macrophages. SHK-1 cells were routinely maintained in L-15 medium (Leibovitz, Gibco) supplemented with 2 mM L-glutamine (Gibco), 40 µM β-mercaptoethanol, and 10% FBS at 18°C under atmospheric conditions. Cells were seeded in six-well plates at a density of 2 × 10⁵ cells per well and allowed to adhere overnight. *P. salmonis* strains were grown for 7–10 days on IFOP-agar plates. Bacterial colonies were harvested and gently resuspended in sterile L-15 medium supplemented with 10% FBS. Suspensions were adjusted to a multiplicity of infection of 100 and added directly to each well. After 1 h of co-incubation with *P. salmonis* at 18°C, gentamicin was added at a final concentration of 50 µg/mL to kill extracellular bacteria and then washed three times with PBS and replaced with fresh culture medium. To evaluate the effect of CRISPRi-mediated repression of NT-associated genes on infection progression, additional assays were performed in 96-well plates seeded with SHK-1 cells at 10 × 10^3^ cells per well; CRISPRi mutants and their respective wild-type controls were grown in IFOP-agar plates and resuspended in L-15 to prepare infection inoculums and infect SHK-1 cells, as previously described. Gentamicin was used to remove extracellular bacteria after 1 h incubation, and IPTG (0.1 mM) was added to L-15 medium for CRISPRi induction. Cellular damage was quantified at different time points using the Invitrogen CyQUANT LDH Cytotoxicity Assay Kit (Thermo Fisher Scientific), which measures lactate dehydrogenase (LDH) release. LDH levels were normalized against the LDH activity of wild-type-infected cells, used as the maximum lysis control (as noted in each figure legend). All infection assays were carried out in biological triplicates.

### RNA extraction and qRT-PCR assays

Total RNA was extracted from (i) SHK-1 cells infected with *P. salmonis* and (ii) *P. salmonis* strains grown in cell-free liquid medium.

For infection-derived RNA isolation, SHK-1 cells were infected with *P. salmonis* wild-type strains (Psal-103 and Psal-104b) previously cultured to stationary phase following the procedure described by Zuñiga et al. (13), but using IFOP broth. Samples were harvested at 48 h post-infection after three PBS washes to remove extracellular bacteria. Three biological replicates (wells) were sampled for each condition. After removing the final PBS wash, cells were directly lysed by adding 1 mL of TRIzol Reagent (Invitrogen) per sample and processed according to the manufacturer’s instructions. After recovery of the aqueous fraction, RNA extraction was performed using the E.Z.N.A. Total RNA Kit I (Omega Bio-tek), following the manufacturer’s instructions.

To confirm that *comEC* expression was successfully induced in *P. salmonis* mutants, qRT-PCR assays were performed after IPTG addition. Cultures were grown in IFOP broth until reaching exponential phase (OD_600_ = 0.5), and IPTG (0.1 mM) was added for 8 h. Untreated cultures were processed in parallel as controls.

In all cases, residual genomic DNA was removed using the TURBO DNA-free Kit (Thermo Fisher Scientific). RNA quantity and purity were assessed with a NanoDrop spectrophotometer, and the absence of genomic DNA contamination was confirmed by PCR using *sdhA*-specific primers without reverse transcriptase (40). For cDNA synthesis, 500 ng of total RNA were reverse transcribed with the iScript cDNA Synthesis Kit (Bio-Rad) using random primers in a 20 μL reaction. Quantitative PCR was performed using the KAPA SYBR FAST qPCR Master Mix on a CFX96 Touch Real-Time PCR Detection System (Bio-Rad). Reactions were run using 2 μL of cDNA per 20 μL of PCR reaction. Gene expression was normalized to the reference gene *sdhA* and analyzed using the 2^⁻ΔΔCt^ method (41). All qRT-PCR primers are listed in Table S2.

## RESULTS AND DISCUSSION

### The *comEC* gene is truncated in all *P. salmonis* genomes sequenced to date

In a previous study, we reported the high-quality sequencing of 55 *P*. *salmonis* genomes, collected over more than 30 years from diverse geographic regions and host species, including representatives of the LF and EM genogroups from Chile, as well as isolates from Canada and Norway (NC genogroups) (16). One key finding, only briefly noted in that work, was the interruption of the *comEC* gene by TEs in all genomes of *P. salmonis* sequenced to date, without exceptions, suggesting an early and selective evolutionary event to disrupt this locus. This is particularly remarkable given that all naturally competent bacteria studied to date encode a functional *comEC* gene, and experimental inactivation of *comEC* typically abolishes transformation capacity (27, 42–44).

Interestingly, in most naturally competent bacteria where NT has been lost or severely impaired by transposon insertions or prophage integrations, the affected loci mainly correspond to regulatory genes, pili components, or elements of the DNA uptake and integration machinery, but rarely to the translocation channel *comEC* (45).

For example, among regulatory elements, the *comK* gene—the master regulator of competence in *Bacillus* (46)—is disrupted by the temperate prophage Φ10403S in various strains of *Listeria monocytogenes* and *L. innocua* (47, 48), both of which possess a nonfunctional competence machinery (47). In addition, an interruption of *comC*—part of the *comCDE* regulatory system—has been reported in a strain of *Streptococcus pneumoniae*, affecting the activation of the competence regulon. Among pili-associated components, *comYC* is disrupted in *Streptococcus pneumoniae*, *S. mutans*, *S. parauberis*, and *Lactococcus lactis*, while the helicase *comFA* is inactivated in some streptococci (48). Likewise, *comGA*—an ATPase essential for pilus assembly—in *Staphylococcus pseudintermedius* (49) and *comC*—a pilus adhesin protein—together with *pilB* (50) and *pilQ* (51) genes in *Acinetobacter baylyi* have been affected by prophage or transposon insertions. Within the group of genes involved in DNA integration and recombination, *comM*—an AAA^+^ helicase in gram-negative bacteria that mediates branch migration during homologous recombination (52)—is the most frequent target of MGEs (45, 51, 53–55). In *A. baumannii*, for example, large resistance islands such as AbaR often insert into *comM*, reducing but not abolishing NT and illustrating an evolutionary trade-off between antibiotic resistance and competence (48).

In contrast, *comEC* disruption by MGEs is rare and has been observed only in a subset (≈25%) of clinical isolates of *S. pyogenes* and *S. pneumoniae*, where transposons of the Tn1207.3/F10394.4 family carry the macrolide resistance operon *mef(A)–msr(D*), abolishing NT (48). Unlike what is observed in *P. salmonis*, the limited distribution of these insertions likely results from antibiotic pressure, promoting the retention of antimicrobial resistance genes (ARGs) instead of counterselection of *comEC*.

**Fig 1:**
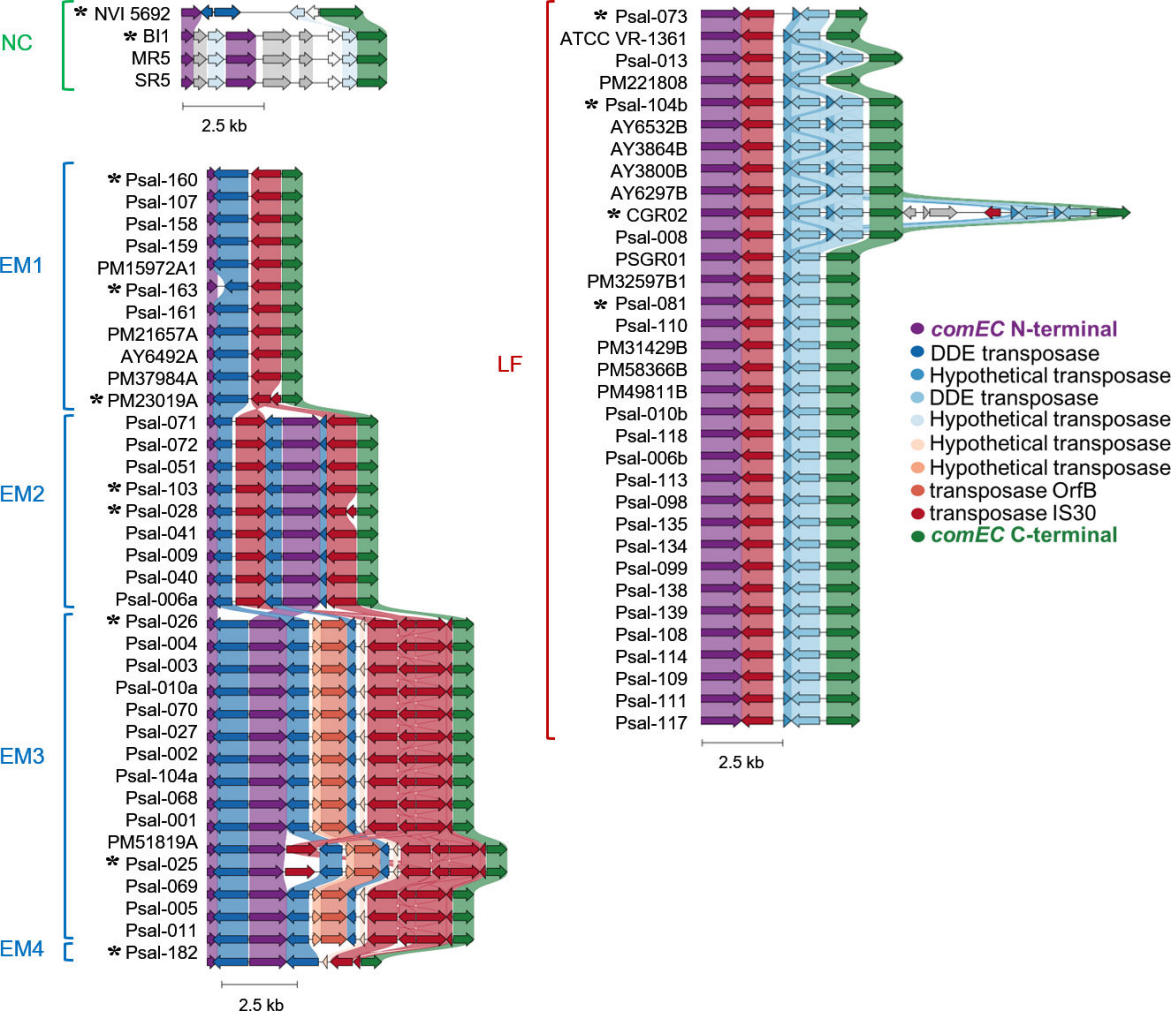
Conserved disruption of the *comEC* CDS in *P. salmonis*. Comparative synteny of *comEC* loci from all publicly available sequenced *P. salmonis* genomes, showing conserved interruption of the *comEC* CDS by TEs across genogroups. The analysis reveals that *comEC* disruptions follow distinct, genogroup-specific architectures, indicating that the structure and position of transposon insertions are conserved within but differ between the phylogenomic subgroups. The remaining *comEC* sequence is shown as N-terminal (purple) and C-terminal (green) fragments; other arrows represent transposase genes, color-coded by homology in Clinker. Shading indicates nucleotide identity between homologous regions (threshold = 0.3). Asterisks mark loci displaying all distinct *comEC*-truncation architectures identified. Figure S1 shows this comparison using only the asterisk-marked loci, and Fig. S2 reports their Pfam domain conservation at the amino acid sequence level (DUF4131, Competence, Lactamase_B).

To better characterize the interruption of *comEC* CDS, we thoroughly examined these loci across all publicly available *P. salmonis* genomes. In both closed and draft assemblies, the locus is consistently interrupted by multiple, distinct TEs inserted at non-identical positions, yielding fragmented and partially eroded CDS. Consequently, no strain harbors an intact *comEC* ORF, and there is no single consensus. Long internal deletions, frameshifts, and premature stop codons are frequent, and the canonical competence domain is absent in some subgroups. Interestingly, sequence alignments revealed a high conservation of TE types and copy numbers within genogroups and subgroups, along with local synteny patterns that defined a characteristic architecture for each group. In total, we identified 14 distinct locus architectures. Pfam domain analysis identified at least three conserved domains—DUF4131, Competence, and Lactamase_B—yet the Competence domain is entirely absent in the EM1 subgroup at the sequence level (Fig. S2). Figure 1 presents a homology-guided gene-cluster comparison generated with Clinker (56), including all sequenced genomes, alongside the phylogenomic classification proposed by Schober et al. (16). These analyses reinforce that *comEC* interruption is deeply rooted in the evolutionary history of *P. salmonis*.

In this sense, the widespread interruption of this gene in *P. salmonis* can be interpreted in the context of the intragenomic conflict model proposed by Croucher et al. (48), recently expanded by Mazzamurro et al. (51) and Vesel et al. (45), which describes antagonistic co-evolution between MGEs and NT-related loci. Under this view, NT could serve as a chromosome-curing mechanism that can remove deleterious MGEs by homologous recombination, whereas MGEs evolve counterstrategies to prevent their loss, such as insertion into competence loci, regulatory repression, or expression of transformation-inhibiting factors (45).

The recurrent and genogroup-specific insertion of distinct transposases within *comEC* in *P. salmonis* is consistent with this model, and the accumulation of TEs: 10%–20% of total CDS in *P. salmonis* are transposases according to Schober et al. (16). Its abundant transposase content and limited HGT between genogroups (16, 48) are consistent with a tendency toward genetic isolation, in which the accumulation of TEs may not be effectively counterbalanced by DNA exchange through NT, for example.

Aligned with this view, despite the extensive use of florfenicol and oxytetracycline in Chilean salmon farming (9, 57, 58), *P. salmonis* appears largely refractory to the acquisition of ARGs, lacking the florfenicol resistance determinants that are widespread in the surrounding microbiota (9, 57, 59–61) and showing only a single report of oxytetracycline efflux pumps likely acquired through HGT (62). While these observations do not allow causal inferences, they are compatible with a genomic landscape shaped by long-term limitations in DNA uptake and genetic exchange.

### A subset of NT-related genes is transcriptionally responsive during infection

Intriguingly, despite the conserved interruption of *comEC*, *P. salmonis* genomes retain multiple predicted genes associated with competence-related functions (Table S4), including annotations such as *comEA*, *comFB*, *comM*, *comL*, and *dprA*. To explore their conservation at the sequence level, we used the Psal-103 and Psal-104b sequences as references for pairwise BLASTp comparisons. In this regard, all NT-related genes exhibited high nucleotide and amino acid identity and coverage between both strains, highlighting their strong conservation across Chilean genogroups (Table 2). When Psal-104b sequences were used as queries against the NCBI ClusteredNR protein database as a genomic proxy for *P. salmonis*, all gene products showed only moderate amino acid identity (typically 40%–60%), mainly with annotated proteins from environmental and aquatic-associated Gammaproteobacteria (also shown in Table 2).

**TABLE 2:** High conservation of NT-related annotations in LF and EM *P. salmonis* genogroups, and sequence divergence with homologs from non-*P. salmonis* bacteria**^*a*^**

Product	Locus tag (GenBank)	CDS and protein ID/cover % (Psal-103 against 104b)	Representative of non-*Piscirickettsia* BLASTp matches against Psal-104b (ID/cover %, GenBank accession)
*comFB*	Psal-103:Psal103_00836 (QGP42219.1) Psal-104b: Psal104b_02435 (UOX25998.1)	CDS: 96/100% Protein: 100/100%	Desulfovibrionales bacterium (44/82%, MDK2956962.1); *Pleionea litopenaei* (46/82%, WP_309202922.1); Seawater Gammaproteobacteria (47/82%, NVJ60226.1); *Aliikangiella* marina (44/82%, WP_142888353.1) *Haliovirga abyssi* (44/82%, WP_307904563.1) *Permianibacter fluminis* (42/62%, WP_172246199.1) *Maridesulfovibrio* sp. (38/61%, WP_320172054.1)
*comL*	Psal-103:*bamD* (QGP43286.1) Psal-104b: *BamD* (UOX24917.1)	CDS: 95.5/99.6% Protein: 96.5/99.6%	Soil Gammaproteobacteria (51/90%, MDF2940624.1); Coxiellaceae bacterium (42/93%, QLH43211.1); *Facilibium subflavum* (43/93%, WP_119342651.1); *Methylocaldum* sp*.* (43/91%, WP_347257417.1); *Thiohalobacter* sp*.* (42/89%, WP_297528073.1) Spongiibacteraceae bacterium (41/93%, MFT3929819.1)
*dprA*	Psal-103:Psal103_03214 (QGP44521.1) Psal-104b: Psal104b_00065 (UOX23705.1)	CDS: 97.8/100% Protein: 98.4/100%	*Legionellales bacterium* (45/97%, MBB71814.1); Methylococcaceae bacterium (46/97%, MEE9337534.1) *Thiolinea* sp. (46/98%, HPY39270.1) *Methylophaga* sp. (44/96%, MBL1321358.1) *Candidatus* Thiodiazotropha sp. (46/96%, MEW8628119.1) *Candidatus* Kentron sp. (45/98%, VFK67218.1) *Candidatus* Competibacter sp. (45/97%, HRW66172.1)
*recA*	Psal-103:*recA* (QGP43128.1) Psal-104b: *recA* (UOX25422.1)	CDS: 97.8/100% Protein: 99.4/100%	Legionellaceae bacterium (80/95%, MCC5792894.1); Coxiellaceae bacterium (81/94%, HYF98132.1); Chromatiales bacterium (79/95%, MDH3946034.1) *Thiothrix* sp*.* (80/96%, WP_002710023.1) Thiohalomonadales bacterium (80/94%, MFV1983620.1) Thioalkalispiraceae bacterium (80/94%, MGD8784215.1) Thiotrichales bacterium (77/94%, HID82103.1) *Sulfuriflexus mobilis* (75/98%, WP_126455699.1) Francisellaceae bacterium (76/97%, MBT4963857.1)
*comEA*	Psal-103:*comEA* (QGP43645.1) Psal-104b: *comEA* (UOX24559.1)	CDS: 97.1/95.2% Protein: 97.4/95.2%	Gammaproteobacteria bacterium (63/72%, MBI3186562.1) Vibrio gazogenes (50/89%, WP_072960937.1) *Vibrio sinus* (60/75%, WP_250613797.1) *Rickettsiella* sp. (46/92%, WP_071662945.1) *Pantoea* sp. (55/75%, WP_313654423.1) *Dokdonella* sp. (55/77%, WP_291225621.1) *Methylomonas* sp. (54/81%, MBS3963912.1)
*comM*	Psal-103:*comM* (QGP44472.1) Psal-104b: *comM* (UOX23752.1)	CDS: 96.4/100% Protein 97.8/100%	Marsh Gammaproteobacteria (60/99%, MEJ2179078.1); *Agitococcus lubricus* (60/99%, WP_107864189.1) *Rudaea cellulosilytica* (59/100%, WP_018971892.1); *Nitrosomonas* sp. (58/99%, WP_046850608.1) *Nitrosococcus oceani* (59/99%, WP_002809266.1) Thiotrichales bacterium (58/99%, MCF6775652.1) *Spartinivicinus marinus* (58/99%, WP_180568461.1) Methylococcales bacterium (57/100%, MBT4838966.1) *Methylophaga* sp. (58/99%, HET8808534.1) *Sedimenticola* sp. (58/99%, WP_428609427.1)

^*a*^ The table shows the annotated gene products, CDS and protein identity and coverage between *P. salmonis* Psal-103 (EM-90-like) and Psal-104b (LF-89-like), as well as the closest non-*P. salmonis* homologs identified by BLASTp, including identity percentage and, alignment coverage (ID/cover %) and GenBank accession numbers using Psal-104b as reference.

**Fig 2:**
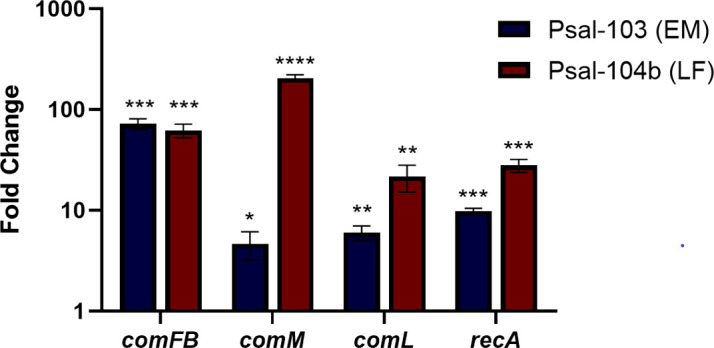
Relative expression of selected NT-related genes of *P. salmonis* during infection of SHK-1 cells. Infections were performed with *P. salmonis* strains Psal-103 and Psal-104b. Fold changes were calculated relative to the bacterial inoculum used for infection. At 48 h post-infection, monolayers were washed to remove extracellular bacteria, and total RNA was extracted. Gene expression was quantified by RT-qPCR using gene-specific primers and the 2^–ΔΔCt^ method, with *sdhA* as the normalization gene. All analyzed genes (*comFB, comL, recA,* and *comM*) were upregulated during infection. Bars represent fold change values (2^–ΔΔCt^) shown as mean ± 95% CI from three biological replicates. Statistical analyses were performed on ΔΔCt values using a one-sample *t*-test against 0 (corresponding to a fold change of 1). *P* < 0.0001 (****); *P* < 0.001 (***); *P* < 0.01 (**); *P* < 0.05 (*).

Although these levels of homology reflect considerable evolutionary divergence, the presence of conserved domains suggests that key structural or functional features may still be preserved, potentially retaining biologically relevant roles even if these are distinct from natural competence pathways, which underscores the importance of further functional characterization of these determinants. To determine whether these genes remain transcriptionally active during infection, we quantified their expression in SHK-1-infected cells relative to bacteria grown on cell-free medium. Under these conditions, *comFB*, *comL*, *comM*, and *recA* were all upregulated during infection, with expression levels markedly higher in Psal-104b than in Psal-103. Specifically, *comFB* increased 72-fold in Psal-103 and 62-fold in Psal-104b; *comM*, 4.6-fold in Psal-103 and 205-fold in Psal-104b; *comL*, 6.0-fold in Psal-103 and 21.7-fold in Psal-104b; and *recA*, 9.8-fold in Psal-103 and 27.9-fold in Psal-104b (Fig. 2). We did not get amplification for *dprA* and *comEA* from the samples corresponding to infected SHK-1 cells. These results indicate that some NT-related genes remain transcriptionally active during host interaction, despite the early and conserved inactivation of *comEC*.

### Heterologous expression of functionally validated *comEC* homologs impairs *P. salmonis* growth

Given that *P. salmonis* retains multiple genetic determinants associated with natural competence, including those involved in the biosynthesis and regulation of T4P, many of which are conserved and transcriptionally active during infection (13, 34), we aimed to evaluate the physiological consequences of heterologous express a functional copy of the missing *comEC* gene. For this purpose, we cloned *comEC* homologs from naturally competent bacteria such as *L. pneumophila* and *V. cholerae* into IPTG-inducible expression cassettes and integrated them into the *P. salmonis* chromosome via the mini-Tn7 system. As mentioned before, in this step, we also included the *comEC* homolog from *Piscirickettsia litoralis* Y2 (63). Although this isolate, recovered from Hawaii, is not a formally recognized species, we found that its draft genome shares high nucleotide identity with *Piscirickettsia salmonis* (ANIb ~75%, data not shown) and contains an intact *comEC* CDS without transposase interruptions, making it an attractive candidate for comparison.

**Fig 3:**
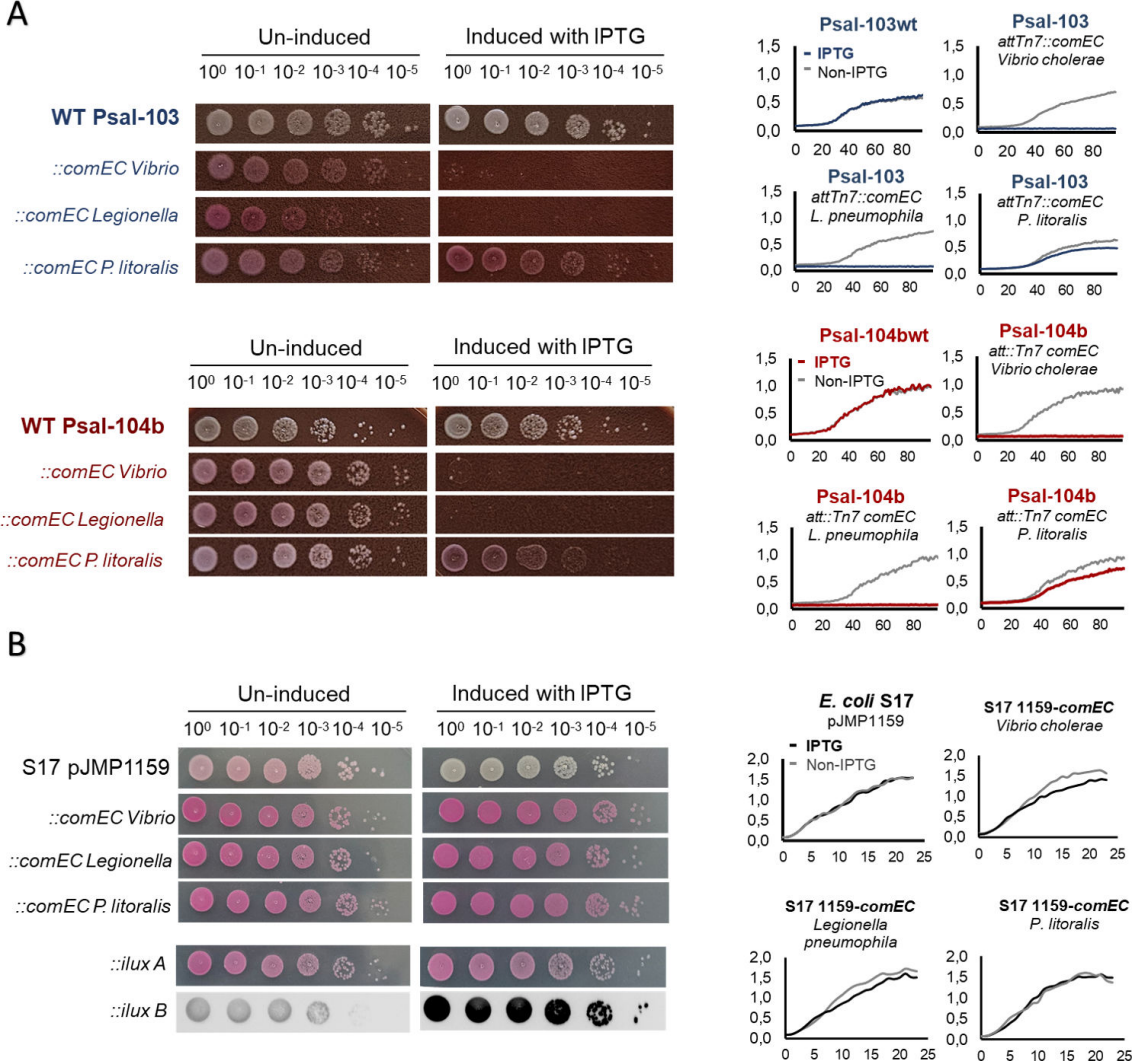
Growth and viability upon heterologous *comEC* expression in *P. salmonis* and *E. coli*. (**A**) Spot-dilution assays and growth curves of *P. salmonis* Psal-103 (top panels) and Psal-104b (bottom panels) carrying IPTG-inducible constructs encoding *comEC* homologs from *Vibrio cholerae*, *Legionella pneumophila*, or *P. litoralis*. Constructs were chromosomally integrated using the pJMP1159 (R6K) mini-Tn7 delivery vector. Wild-type strains were assayed in parallel and included as controls. (**B**) Equivalent assays in *E. coli* donor strains carrying the same IPTG-inducible pJMP1159-derived plasmids used for *P. salmonis* complementation in panel A, prior to chromosomal delivery to *P. salmonis*. For all experiments in panels A and B, gene expression was induced with 0.1 mM IPTG. The original pJMP1159 (i.e., carrying an *rfp*-CRISPRi) vector and a Plac-regulated ilux reporter are shown as IPTG-induction controls for *E. coli. ::ilux* A indicates the direct photographic register of the spots, and ::*ilux* B depicts the luminescent signal emitted by the same spots from ::ilux A.

Interestingly, IPTG-induced expression of the *comEC* homologs from *L. pneumophila* and *V. cholerae* resulted in severe growth inhibition in both Psal-103 and Psal-104b strains, as revealed by growth curves and serial spot dilution plating on solid medium (Fig. 3A). In contrast, expression of the *P. litoralis* Y2 homolog did not produce the same effect on growth under equal conditions. RT-qPCR analysis confirmed that all constructs were transcriptionally activated upon IPTG exposure (Fig. S3), supporting the notion that the observed growth defects arise from the induction of these specific *comEC* variants. An interesting aspect to note about these results is that the growth arrest observed following heterologous expression of *comEC* homologs from *L. pneumophila* and *V. cholerae* does not occur in *E. coli* S17 λ pir: the bacterium used as a donor to transfer *comEC* genetic constructs to *P. salmonis* (Fig. 3B). This finding is remarkable since, in S17 λ pir, the pJMP1159 plasmid carries multiple copies of the *comEC* gene (i.e., acting as an independent replicon from its R6K origin), whereas in *P. salmonis,* it is introduced as a single chromosomal copy via the mini-Tn7 system.

These findings suggest that the expression of fully functional *comEC* homologs (from *Legionella pneumophila and Vibrio cholerae*) imposes a significant fitness cost on *P. salmonis*, which could help explain the sustained selective pressure inferred by the interruption of this gene in all sequenced isolates of *P. salmonis*. On the other hand, the *P. litoralis* homolog appears to be efficiently expressed and does not impair growth under induced conditions. This difference cannot be strictly associated with phylogenetic distance, as both *P. litoralis* (draft) and *L. pneumophila* are closely related to *P. salmonis.* However, the functionality of *P. litoralis* ComEC in *P. salmonis* remains to be evaluated, although any interpretation of this *comEC* annotation should be made with caution, as this strain has not been recovered for laboratory work since its initial report (63). Importantly, our data do not support conclusions regarding the function or fitness effects of an ancestral *P. salmonis* ComEC, but rather indicate that the expression of certain functionally validated *comEC* homologs is poorly tolerated in this bacterium.

### Functional analysis of NT-related genes in *P. salmonis* via CRISPRi repression reveals strain-specific phenotypes between Psal-103 and Psal-104b

To further investigate the essentiality of putative NT-associated genes, we generated conditional knockdown mutants of Psal-103 and Psal-104b using an IPTG-inducible dCas9-based CRISPRi system, aiming to evaluate their impact on growth in cell-free medium and pathogenesis in the SHK-1 macrophage-like cell infection model. Six genes typically involved in DNA uptake and recombination were individually targeted, including *comFB*, *comL* (*bamD*), *comEA*, *comM*, *dprA*, and *recA*.

**Fig 4:**
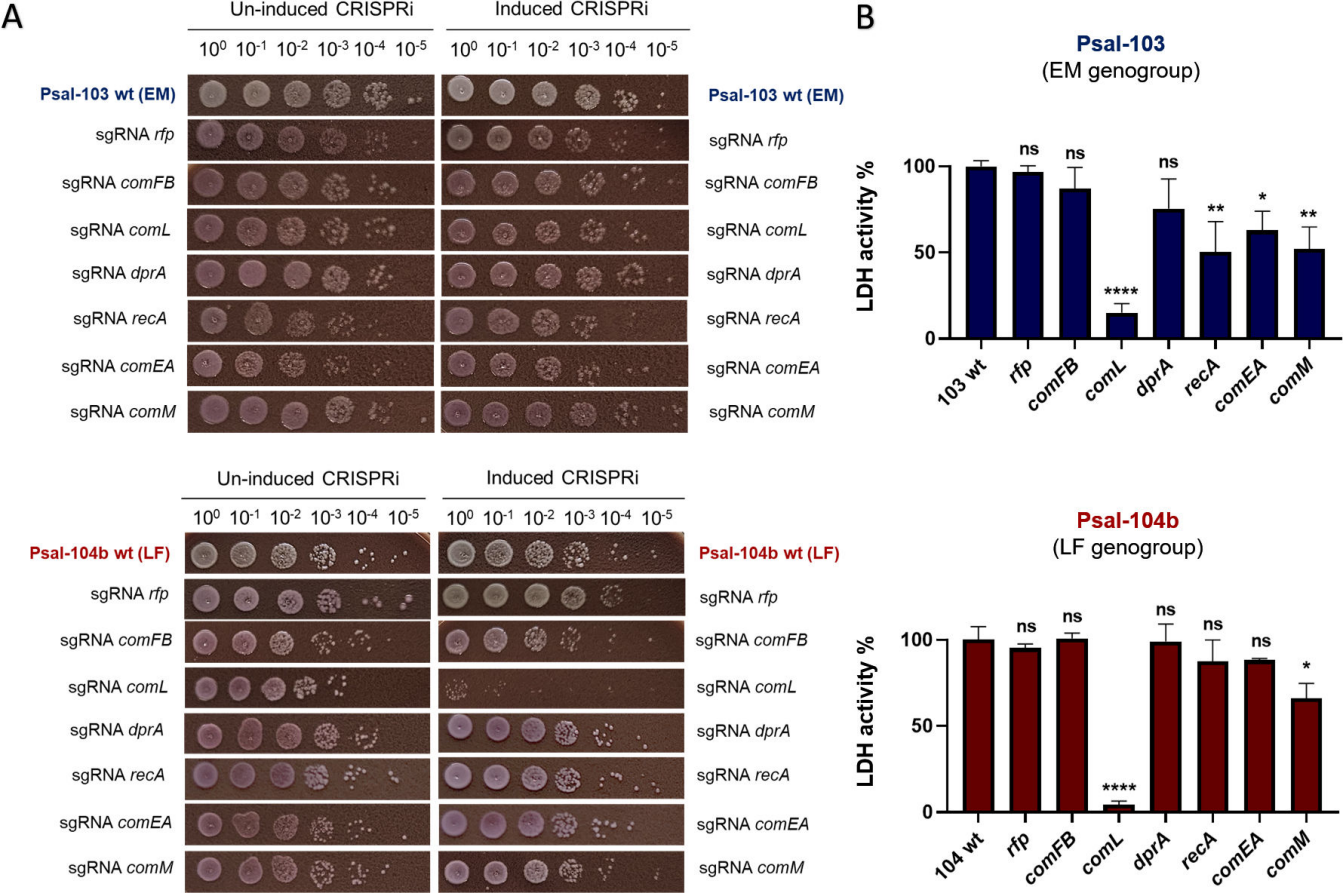
CRISPRi-based functional interrogation of NT-related genes in *P. salmonis*. (**A**) Spot-dilution viability assays of CRISPRi knockdown mutants in *P. salmonis* Psal-103 (EM; top) and Psal-104b (LF; bottom). Each strain harbors a mini-Tn7 chromosomal insertion encoding *dCas9* and a gene-specific *sgRNA*, both under control of the IPTG-inducible *Plac* promoter. Targeted genes included *comFB*, *comL*, *dprA*, *recA*, *comEA*, and *comM*. Serial 10-fold dilutions were plated on IFOP agar in the absence (left panels) or presence (right panels) of 0.1 mM IPTG. A control strain expressing an sgRNA against the rfp reporter was used as a control of the CRISPRi system. (**B**) Cytopathic damage produced by the same CRISPRi mutants in SHK-1 cells under CRISPRi-inducing conditions (0.1 mM IPTG). LDH release was measured at 14 dpi, and levels are expressed relative to the respective wild-type strain (100% LDH activity). Statistical significance was assessed by one-way ANOVA followed by Dunnett’s multiple comparison test. *P* < 0.05 (*), *P* < 0.01 (**), *P* < 0.0001 (****), ns = not significant. The time course of infection progression (4, 7, 11, and 14 days post-infection) is shown in Fig. S5. Psal-103 WT and Psal-104b WT (with and without IPTG) captures in panel A are the same as those used in Fig. 3A.

Viability dilution assays on IFOP agar (Fig. 4A) and growth curves in IFOP broth (Fig. S4) showed that CRISPRi-mediated repression of *comL* severely impaired the viability of Psal-104b (LF genogroup) under cell-free culture settings. In contrast, repression of *comL* in Psal-103 (EM genogroup), as well as repression of any of the other targeted genes in both strains, did not detectably affect growth, suggesting that these genes are not essential for proliferation in axenic media and that *comL* displays genogroup-specific essentiality.

Interestingly, *comL* was originally identified in *Neisseria gonorrhoeae* as a lipoprotein specifically required for DNA uptake during NT, firmly establishing its role within the competence machinery of this species (64). Subsequent studies revealed that *comL* corresponds to *bamD*, a gene encoding a highly conserved periplasmic lipoprotein that forms part of the β-barrel assembly machinery (BAM) in most gram-negative bacteria (65). This complex is responsible for the insertion and correct folding of outer membrane proteins. Among its five subunits (BamA–E), where BamA serves as the core and universally essential component, only BamD is generally indispensable for proper BAM function, and in many species, its loss results in lethality comparable to that caused by the absence of BamA (65–69). Together, these findings suggest that while *bamD* is essential for viability *in* Psal-104b, its role in Psal-103 may be potentially modulated by various factors, including functional redundancy, differences in trans-acting regulatory factors, genogroup-specific envelope stress responses, or limitation to host-associated contexts.

To interrogate all of these CRISPRi mutants in an infection setting, SHK-1 cells were infected in the presence of 0.1 mM IPTG to induce gene repression, and cytopathic damage was quantified by LDH release relative to the corresponding wild-type strains (Fig. 4B). As expected, repression of *comL/bamD* in Psal-104b resulted in almost complete loss of cytopathic effect, consistent with the lethality observed in cell-free media. Interestingly, repression of *comL* also markedly reduced cytopathic activity in Psal-103 (approximately 85% relative to the wild-type strain), indicating an infection-specific requirement for this gene, despite its non-essentiality in axenic media.

Repression of *comM* significantly reduced cytopathic effect in both strains, pointing to a conserved role in infection-associated processes. On the other hand, repression of *comFB* in Psal-104b *P. salmonis* strains caused a modest delay in infection progression, but none in Psal-103 (Fig. S5). Although *comFB*-like proteins are consistently found within competence loci across diverse bacteria, they remain mainly uncharacterized. Recent studies suggest they may participate in cyclic dinucleotide-mediated signaling pathways, potentially linking them to processes such as biofilm formation and persistence (70). These parallels point to possible roles beyond transformation in *P. salmonis.*

In Psal-103, *recA* repression led to a significant reduction in cytopathic effect, whereas no such impact was observed in Psal-104b. *recA* encodes a central recombinase essential for homologous recombination and DNA repair, crucial for genome integrity and survival under stress (71, 72). Similar attenuated phenotypes have been reported in *recA* mutants of diverse bacterial pathogens, highlighting its pleiotropic roles in virulence, mutagenesis, persistence, or antibiotic resistance (71, 73, 74). This genogroup-specific phenotype is consistent with previous RNA-seq data showing lower induction of the recA–recX operon in EM strains during infection (75), which may reflect differences in DNA repair and stress-response dynamics during host interaction.

For both *comEA* and *dprA*, no detectable phenotypic effects were observed in Psal-104b (LF). In Psal-103 (EM), repression of these genes was associated with a reduction in cytopathic effect, although only *comEA* showed a modest but statistically significant decrease in LDH activity during infection. This observation aligns with the canonical role of *dprA* in NT, typically activated during late competence to mediate the transition from DNA uptake to homologous recombination. Although *dprA* has been implicated in virulence in *Streptococcus pneumoniae* (76–78), this appears to represent a notable exception rather than a widespread adaptation across bacteria. Another example of alternative functions for NT-related genes occurs in *S. pneumoniae* and *S. mutans*, where the alternative sigma factor ComX (also known as SigX)—together with the upstream two-component system ComDE—regulates not only the expression of late competence genes but also modulates stress responses, quorum sensing, bacteriocin production, biofilm formation, and virulence-associated pathways (79–81). The *comM* gene of *S. pneumoniae* (unrelated to the AAA^+^ ATPase *comM* of gram-negative bacteria), in which the product is a membrane-associated immunity protein expressed early during competence (82) and protects competent cells from self-inflicted lysis caused by murein hydrolases (such as CbpD, LytA, and LytC) released to kill non-competent siblings during the pneumococcal fratricide process (83, 84). Deletion of *comM* renders even competent cells susceptible to lysis and leads to intense DNA-mediated clumping, whereas its overexpression inhibits cell division by blocking both the initiation and the final constriction of the cytokinetic ring (82). These observations highlight how regulators and effectors originally linked to DNA uptake can become embedded within broader cellular programs.

Examples from other species further illustrate the functional versatility of NT-related genes. In *Listeria monocytogenes*, the competence regulon can be reactivated during intracellular infection, promoting phagosomal escape and virulence independently of transformation (47). In *Bacillus subtilis*, *comGA* not only assembles the DNA uptake machinery but also interacts with RelA, the (p)ppGpp synthetase, thereby inducing growth arrest during competence (85). Likewise, *comEB* functions as a dCMP deaminase involved in both NT and nucleotide metabolism (86).

While the demonstrated role of *comL/bamD* in natural competence appears to be restricted to *N. gonorrhoeae*, its function alongside BamA as part of the BAM complex is broadly conserved and essential for the viability of many gram-negative bacteria. Accordingly, disruption of this complex has emerged as an actively investigated therapeutic strategy against gram-negative pathogens (87–90). In *P. salmonis*, the pronounced fitness impairment observed upon *comL/bamD* repression supports a critical role for this system in bacterial physiology. This observation is also consistent with previous omics studies reporting infection-associated changes in outer membrane protein transport and envelope-related pathways (13, 91). However, our conclusions are based on phenotypic assays and do not directly address the molecular mechanisms underlying BAM complex dysfunction in this species, which will require further investigation.

Deeper mechanistic analyses may help clarify how perturbation of this system affects outer membrane protein biogenesis and envelope-associated processes, and whether these changes have physiological or therapeutic relevance.

Finally, the differential phenotypes observed in some cases throughout this study in Psal-103 and Psal-104b—used here as proxies for the major Chilean genogroups—add to a broad body of previously reported genogroup-associated differences, including distinctive colony morphotypes (mucoid in EM and “sticky” in LF strains), differential quinolone susceptibility (16, 92), documented contrasts in disease progression and clinical outcomes (93), variable vaccine effectiveness (94, 95), among others (19, 75, 96, 97). Together, these observations underscore the importance of systematically including representatives of both Chilean genogroups in experimental design for future studies, epidemiological surveillance, and in the development of genogroup-aware management strategies.

## SUPPLEMENTAL MATERIAL

Supplementary figures spectrum.03173-25-s0001.docx Figures S1 to S4.

Supplementary tables spectrum.03173-25-s0002.xlsx Tables S1 to S4.

ASM does not own the copyrights to Supplemental Material that may be linked to, or accessed through, an article. The authors have granted ASM a non-exclusive, world-wide license to publish the Supplemental Material files. Please contact the corresponding author directly for reuse.
