# Supplementary material for: Genomic and functional dissection of natural transformation-related genes in Piscirickettsia salmonis
Source: Microbiol Spectr. 2026 Feb 4;14(3):e03173-25. doi: 10.1128/spectrum.03173-25 (PMC12955456; doi:10.1128/spectrum.03173-25)
Supplement: Supplementary figures — Figures S1 to S4. [file spectrum.03173-25-s0001.docx]

**Figure S1**. Syntheny analysis of all distinctive *comEC*-loci archetypes in *P. salmonis*. The remaining *comEC* CDS are depicted in purple (N-terminal) and green (C-terminal); all other CDS correspond to transposase or non-identified sequences. The analysis reveals that *comEC* disruptions follow distinct, genogroup-specific architectures, indicating that the structure and position of transposon insertions are conserved within but differ between the phylogenomic subgroups.


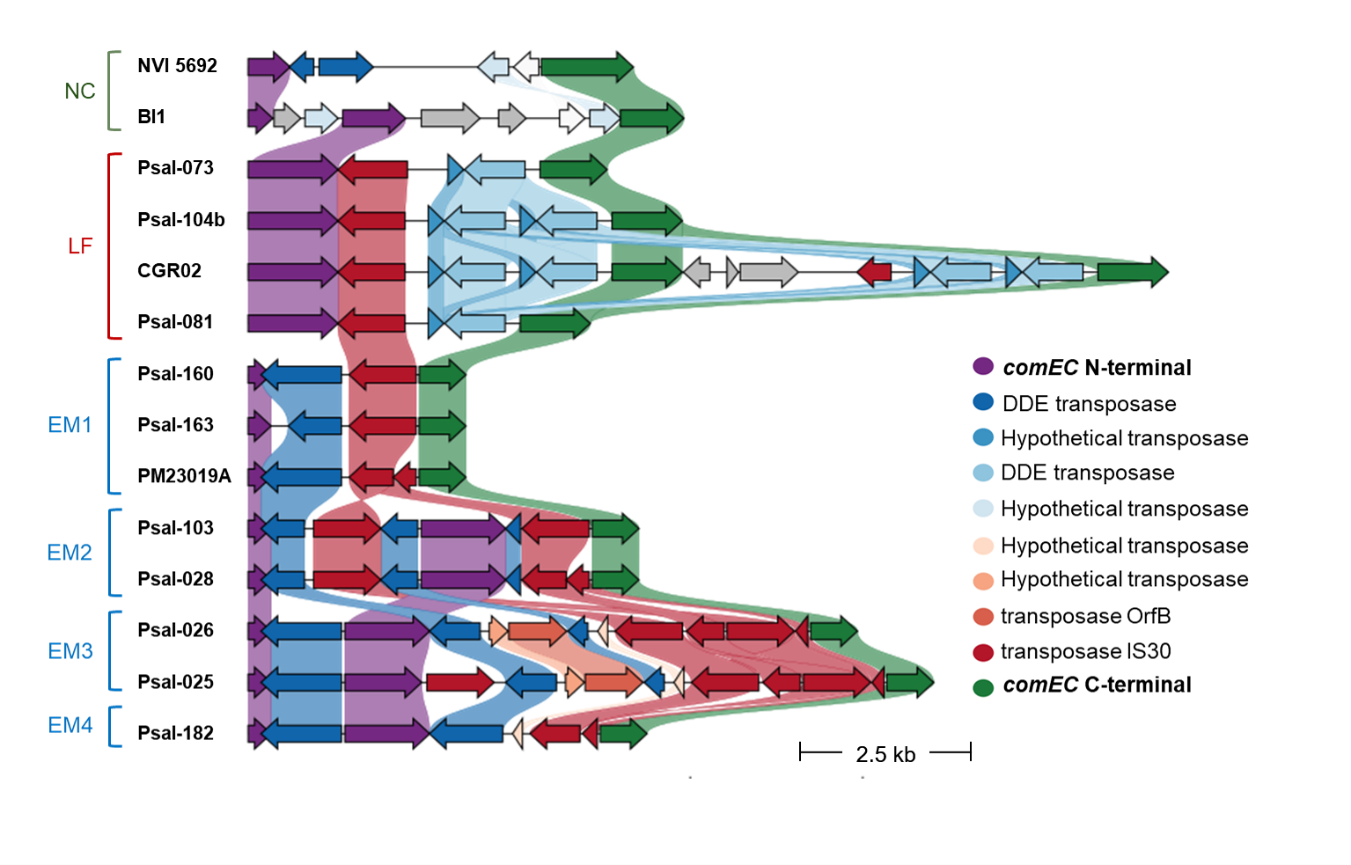


**Figure S2**. Aligned concatenated ComEC coding sequences showing overall amino acid conservation. Purple “XXX” indicate the insertion site for transposases in each locus. Blue, red and green residues were identified by Pfam analysis as DUF4131, Competence and Lactamase_B domains, respectively.


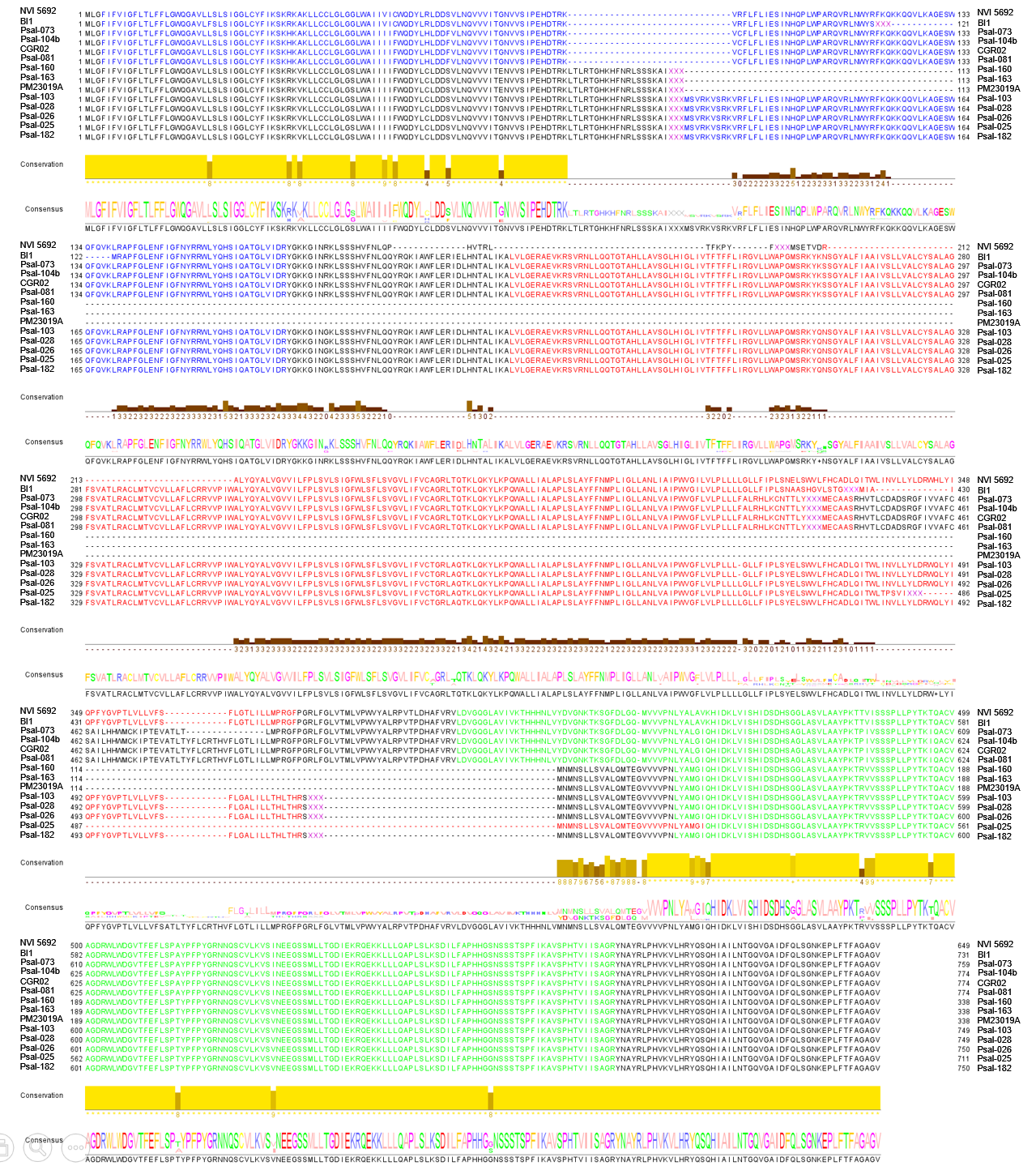


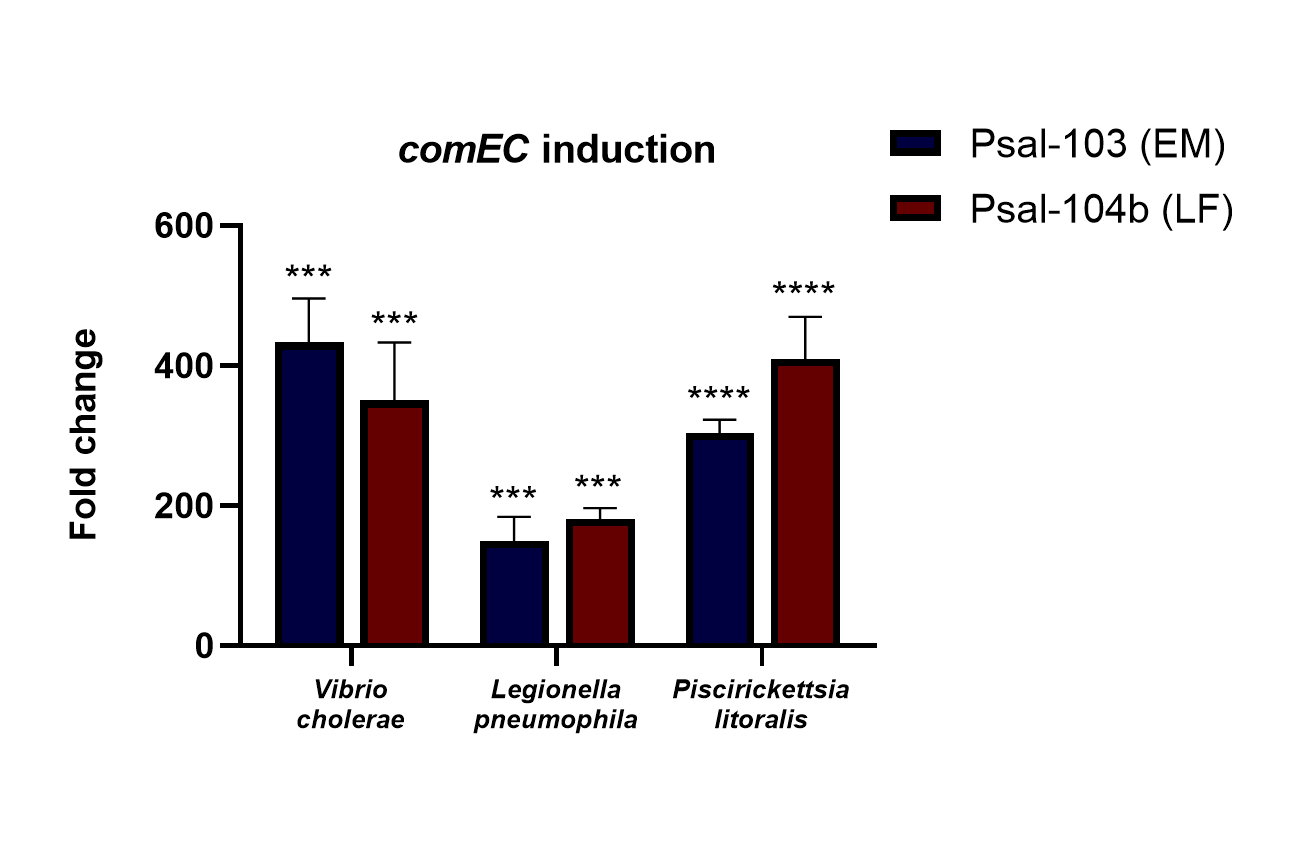
**Figure S3. Expression levels of *comEC* homologs in *P. salmonis* strains Psal-103 (red bars) and Psal-104b (blue bars).** Strains express *comEC* homologs from *Vibrio cholerae*, *Legionella pneumophila*, and *Piscirickettsia litoralis* under the control of the IPTG-inducible *Plac* promoter. Induction was performed during exponential growth to minimize toxic effects associated with *comEC* expression. Gene expression was quantified by RT-qPCR using gene-specific primers and the 2^–ΔΔCt method, with *sdhA* used as the normalization gene. The results confirm transcriptional activation of *comEC* homologs under IPTG-inducing conditions (0.1 mM IPTG) in all genetic backgrounds. Statistical significance was assessed on ΔΔCt values using a one-sample *t* test against 0 (corresponding to a fold change of 1). *P* < 0.0001 (); *P* < 0.001 (*); *P* < 0.01 (); *P* < 0.05 (*).

**Figure S4. Growth curves CRISPRi knockdown of Psal-103 and Psal-104b strains targeting NT-related genes.** Bacterial growth was monitored for wild-type and CRISPRi derivatives of *P. salmonis* strains Psal-103 (blue) and Psal-104b (brown) harboring sgRNAs against *comFB*, *dprA*, *comL*, *recA*, *comEA*, or *comM*. Cultures were inoculated into IFOP broth at an initial OD600 of 0.02 and incubated statically at 18 °C for 96 h. Optical density at 600 nm was recorded at regular intervals. Each strain was analyzed under non-induced conditions (gray, no IPTG) and under induced conditions (red, 0.1 mM IPTG; blue, 0.01 mM IPTG). Curves data represent mean values from biological triplicates.


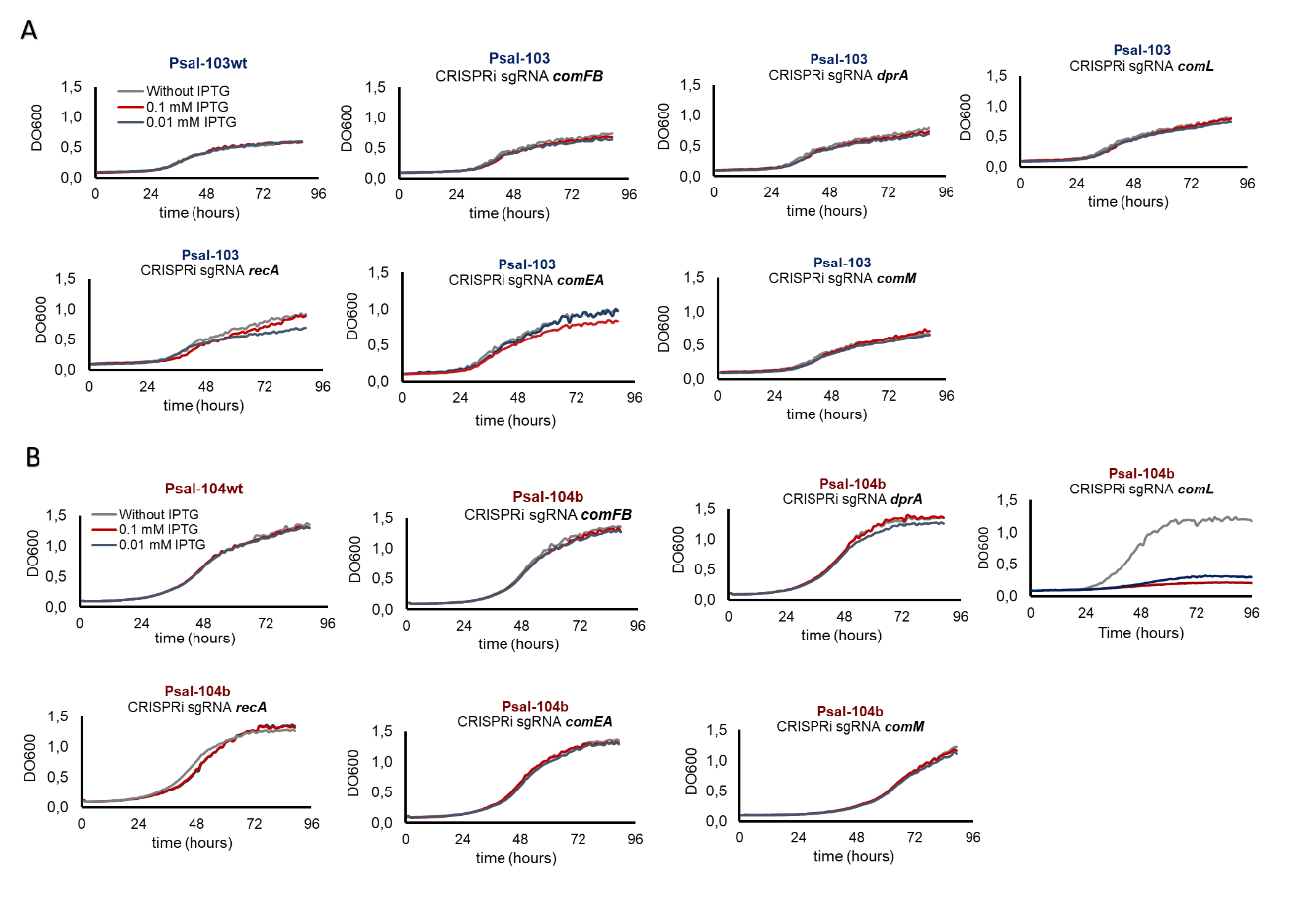


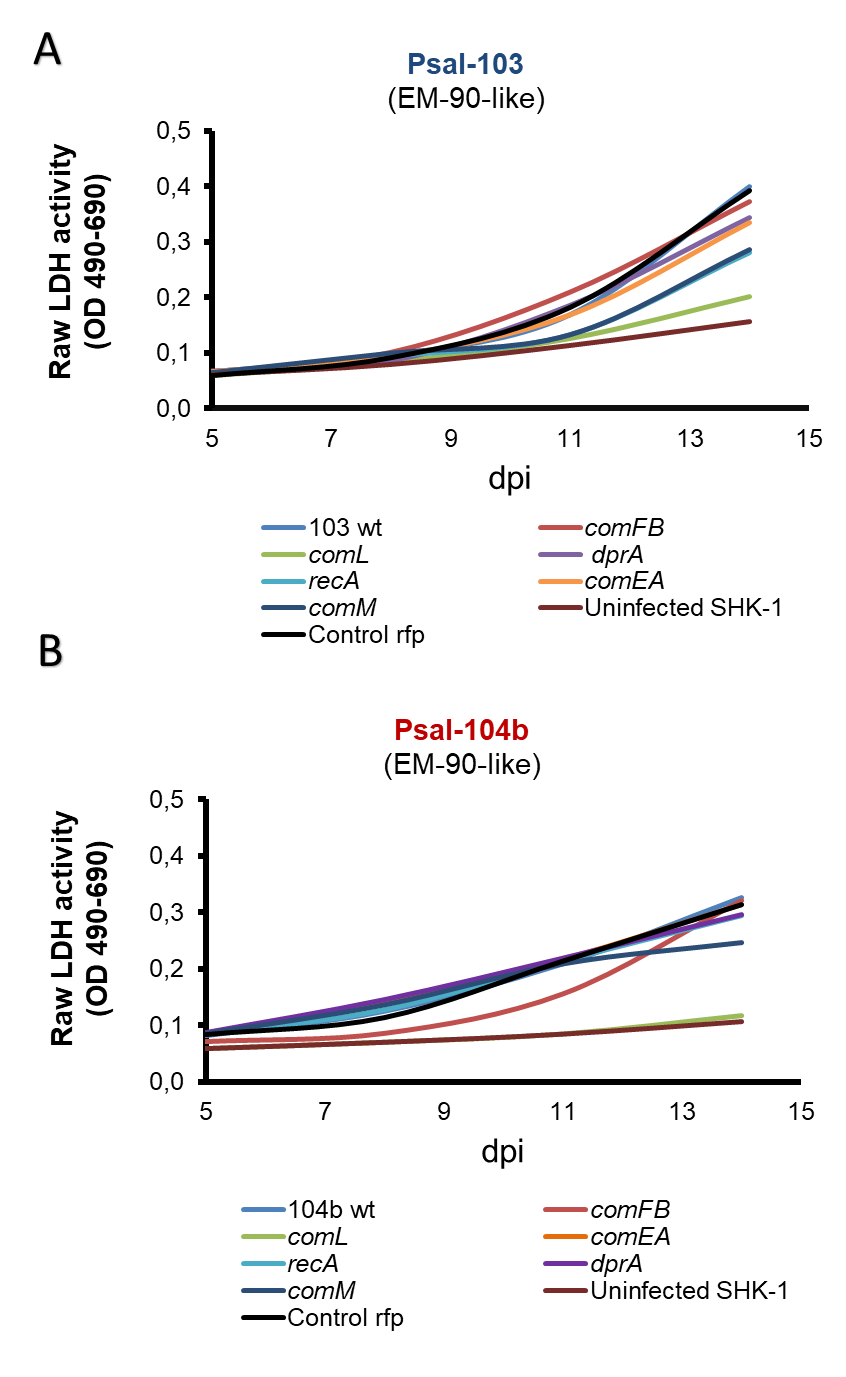
**Figure S5.** Cytopathic effect induced by CRISPRi-mediated knockdown of NT-related genes in *P. salmonis* strains Psal-103 (A) and Psal-104b (B). SHK-1 cells were infected under CRISPRi-inducing conditions (0.1 mM IPTG), and lactate dehydrogenase (LDH) release was measured at 4-, 7-, 11-, and 14-days post-infection (dpi). Mean values for LDH activity (OD 490–690) is shown for *comFB*, *comL*, *comM*, *comEA*, *dprA*, and *recA* knock-down mutants. The respective wild-type strains and an uninfected SHK-1 control are included for comparison.
